# An Ambulatory System for Gait Monitoring Based on Wireless Sensorized Insoles

**DOI:** 10.3390/s150716589

**Published:** 2015-07-09

**Authors:** Iván González, Jesús Fontecha, Ramón Hervás, José Bravo

**Affiliations:** MAmI Research Lab, University of Castilla-La Mancha, Esc. Sup. de Informática, Paseo de la Universidad, 4, 13071 Ciudad Real, Spain; E-Mails: ivan.gdiaz@uclm.es (I.G.); ramon.hlucas@uclm.es (R.H.); jose.bravo@uclm.es (J.B.)

**Keywords:** gait analysis, gait monitoring, wearable sensor, sensorized insole, gait phase detection, fuzzy inference, activity recognition

## Abstract

A new gait phase detection system for continuous monitoring based on wireless sensorized insoles is presented. The system can be used in gait analysis mobile applications, and it is designed for real-time demarcation of gait phases. The system employs pressure sensors to assess the force exerted by each foot during walking. A fuzzy rule-based inference algorithm is implemented on a smartphone and used to detect each of the gait phases based on the sensor signals. Additionally, to provide a solution that is insensitive to perturbations caused by non-walking activities, a probabilistic classifier is employed to discriminate walking forward from other low-level activities, such as turning, walking backwards, lateral walking, etc. The combination of these two algorithms constitutes the first approach towards a continuous gait assessment system, by means of the avoidance of non-walking influences.

## 1. Introduction

Quantitative gait analysis (QGA) is the systematic study of human walking, in terms of kinematics, kinetics, energy expenditure and spatio-temporal parameters useful for the characterization of human movement. Gait analysis is applied in several fields of study for different purposes, such as the assessment of gait pathologies and identification of gait abnormalities [1–3]; or kinesthetic feedback devices and functional electrical stimulation (FES) systems to improve walking or to control the gait cycle of a prosthesis based on gait phase detection [4–8].

FES systems are used for a wide range of gait disabilities (e.g., after brain strokes, spinal cord injuries or neurological diseases), including disabilities found in children (e.g., spastic diplegia or cerebral palsy). Other examples under the scope of QGA are focused on improving feedback in the rehabilitation process at home or in community dwellings through the measurement of motor recovery from several gait parameters. These systems provide continuous data for both the subject who is undergoing rehabilitation therapy and the therapist [9].

Additional uses of QGA in clinical assessment relate to the use of the estimated gait parameters, along with appropriate reasoning techniques to guide physicians to optimal decision making for improving gait through orthotic management. In such cases, QGA can provide objective and progressive data about the gait deviation and functional deficits that must be addressed with a custom orthosis [10].

Another potential use of QGA is associated with the prevention of pressure ulcers in diabetic patients who have peripheral neuropathy, due to the loss of pain sensation and repetitive plantar pressure. Foot force distribution measurement is important in the assessment of diabetic feet [11,12]. In the same vein, Ranu [13] confirms that patients with peripheral neuropathy develop over 60% higher forefoot pressures when compared to normal subjects. Feet are definitely the most frequent area of ulceration in patients hospitalized for diabetes mellitus. In this sense, foot orthosis [14] is the common treatment for plantar pressure relief and to avoid ulceration. However, there are few studies that examine the direct impact of different shape variations of orthotic insoles on plantar pressure for peripheral neuropathy [15].

Moreover, QGA sometimes has relevant requirements that must be fulfilled to make it a powerful tool for diagnosing and monitoring the clinical course of specific disabilities or diseases. For example, the inherent gait variability [16–19] over time is a crucial aspect for diagnosing frailty syndrome and neurodegenerative diseases (such as Parkinson and some types of dementia) or for predicting fall risk. In this case, continuous gait monitoring over time [20–24] is useful to provide reliable analysis and characterization of the gait changes. Furthermore, as in the FES systems presented above, several clinical applications of QGA require a real-time approach to gait monitoring.

In this paper, we introduce a gait phase detection system based on a pair of wireless sensorized insoles. The system enables the detection of five phases of the human gait cycle (loading response (LR), mid-stance (MSt), terminal stance (TSt), pre-swing (PSw) and swing (Sw) [1]) in real-time by means of a gait phase detection algorithm (GPDA). Our GPDA is based on fuzzy logic, with the core system implemented on a smartphone and running in the background. Therefore, all of the processing from sensor signals to gait phases is done by the phone. However, one microcontroller per insole (together with a Bluetooth module) is used as a communication interface between the sensors and the smartphone.

On the other hand, to provide a system insensitive to perturbations caused by non-walking activities, such as standing up or sitting down, we propose a separate stage in our solution consisting of the use of a Gaussian naive Bayes classifier (GNB) to discriminate walking forward from other low-level activities, such as turning, walking backwards, lateral walking, *etc*. In other words, this second step allows us to recognize when the subject is walking forward and to distinguish this functional activity from others.

Once the GNB classifier identifies a walking forward action at a particular time, the system can enable the GPDA to store the estimated gait parameters for further processing and analysis (e.g., to evaluate the quality of the gait in a Parkinson's sufferer over time). With this approach, the conjunction of the GPDA and the GNB classifier is a powerful combination for creating a continuous gait assessment system in controlled environments (e.g., at home or in community dwellings).

The rest of this article is organized as follows. Section 2 provides the related work of existing solutions and sensor systems for QGA, centered on the spatio-temporal dimension of the gait cycle (particularly those focused on gait phase detection). Section 3 then describes the proposed solution's hardware architecture and methodology to detect gait phases during continuous monitoring. This section contains two main subsections, one explaining the gait phase detection algorithm's details (Section 3.2) and the other explaining how the algorithm based on principal component analysis (PCA) and the GNB classifier are used to discriminate walking forward from other low-level activities, guaranteeing a system insensitive to non-walking activities (Section 3.3). In Section 4, we describe the experimental setup and the evaluation results obtained with both algorithms. Finally, Section 5 exposes the conclusion and areas for future work.

## 2. Related Work

Several works have proposed insole-based devices, each with a different arrangement of sensors, as wearable systems for the human gait's spatio-temporal analysis. For example, Crea *et al.* [25] have developed an opto-electric pressure-sensitive cell with dimensions of 12 mm × 12 mm and a height of 5.5 mm. They have built a flexible insole that uses 64 of these pressure-sensitive cells together with an integrated electronic board for high-frequency data acquisition, pre-filtering and wireless transmission to a remote computing unit. One of the advantages of this device is that the sensor placement covers nearly the whole surface of the foot; therefore, the sensing area is large enough to allow an accurate estimate of both the spatial coordinates of the center of pressure (CoP) and the vertical ground reaction force (vGRF). The main disadvantage of this device is that the sensors have a bad linearity at low and high pressure, which will require more complex signal processing to ensure an accurate representation of the pressure.

Crea *et al.* take advantage of the accurate estimation of vGRF and CoP*_y_* in their system and use these biomechanical variables to implement a GPDA based on a single threshold. The gait phase detection algorithm proposed is capable of identifying three gait phases: “Stance 1”, “Stance 2” and “swing”. The relationship between the gait phases detected by their system and the theoretical model of human gait proposed by Perry [1], which has been also used as a reference model in this paper, is shown in Table 1.

Another remarkable study that uses sensorized insoles comes from Bamberg *et al.* [26]. Their work proposes a highly elaborated wearable system named GaitShoe, composed of three orthogonal accelerometers, three orthogonal gyroscopes, four FSRs (force-sensitive resistors), two piezoelectric pressure sensors, two bend sensors (used to analyze flexion during gait) and electric field height sensors to measure the foot clearance. The device is able to collect data unobtrusively, in any environment and over long periods of time. Bamberg *et al.* use it for quantitative gait analysis in healthy and Parkinsonian subjects. The sum of four FSR sensors is utilized to obtain two gait time events: heel-strike (HS) and toe-off (TO). However, instead of using fixed thresholding operations, HS is marked after the local maxima of the sum of FSRs that exceed the previous by more than 0.005 kg; similarly, TO is set at the first time point after the local minima within 0.005 kg of the following point. Besides HS and TO, the GaitShoe estimates foot orientation, velocity and stride length.

Lopez-Meyer *et al.* [27] also rely on the sum of five FSR sensors to estimate HS and TO events. In their case, the proposed methodology adjusts a dynamic threshold based on the pressure behavior of the sum of all five FSR sensors located in a shoe-based wearable. The adjustable threshold algorithm is less sensitive to variability in the subject's weight and gait asymmetry and to intersubject variability (due to the system's evaluation with poststroke subjects with different levels of hemiparesis). Other temporal parameters are obtained beyond HS and TO: gait cycle time, step time, stance, swing, single support and double support.

In [28], Schepers *et al.* present an ambulatory measurement system able to reliably estimate foot placement during walking. Their prototype consists of a pair of orthopedic sandals composed of two six-degrees-of-freedom force/moment sensors beneath the heel and the forefoot and two six-degrees-of-freedom inertial sensors rigidly attached to the force/moment sensors. Analysis of the human gait's spatio-temporal dimension is not this solution's main purpose, although it provides a tool to estimate the heel-strike (HS) and heel-off (HO) time events through its force sensors and the two spatial parameters lateral foot placement (LFP) and stride length (SL), by the integration of its inertial sensor signals. The ambulatory system is compared to a reference optical position measurement system (Optotrak) as a golden standard. The system is able to discriminate between different walking conditions (walking with “eyes open” and with “eyes closed”) with accuracy. In this study, the sensor integration is remarkable, as is the force sensors' ability to define the initial and final conditions that help reduce the drift caused by integration of the accelerometer and gyroscope signals.

A different approach also using inertial sensors and two mobile force plates mounted under instrumented shoes is proposed by Liu *et al.* [29]. In this case, kinematic and kinetic analyses of lower limbs are implemented based on a combination of measurements of 3D segment orientations (calculated from inertial sensors located at the ankle, knee and hip) and the ground reaction force obtained from the force sensors.

The sensorized insoles implemented by Pappas *et al.* [4] have commonalities with our solution, in terms of pursued aims. They offer a real-time gait phase detection system with support for continuous gait monitoring and insensitivity to non-walking activities. These similarities are described in Table 2.

The previous studies described in this section propose insole-based devices that generally rely on threshold methods or fixed state rules to estimate gait parameters or gait phases; however, some cases remain where subjects have extremely light pressure patterns during gait, which result in very low sensor signals. These gait patterns could go undetected by a threshold algorithm or a finite state machine. A smoother GPDA based on fuzzy logic can provide more accurate gait pattern detection [30]. In this regard, each gait phase can be represented as a combination of sensor inputs.

Another different study also focused on gait phase detection by pressure patterns is presented in [31]. In this case, a mixture model composed of Bernoulli distributions is trained using the expectation maximization (EM) algorithm over a time series collection of four pressure sensor inputs during a walking exercise. The mixture model is interpreted in terms of gait phases.

Furthermore, sensorized insoles and shoes have been used to perform tasks other than gait analysis. For example, the study carried out by Edgar *et al.* [32] combines in-shoe sensors (an assembly of five FSRs and a triple-axis accelerometer ADXL330, designed to operate in free living conditions) and a smartphone for automatic recognition of postures and low-level activities, such as walking, sitting, *etc*. Edgar *et al.* assess the identified activities to improve rehabilitation strategies after strokes, providing patients with behavior-enhancing feedback and helping them to drive cortical reorganization to increase their activity levels. This device is also used by Sazonova *et al.* [33]. In their case, the data the footwear system supplies are utilized to estimate energy expenditure (EE). EE prediction is performed as a two-step process, with the first step as classification of postures/activities into one of the four groups: sit, stand, walk and cycle [32]; and the second step as EE prediction that uses the adequate branch of the four regression models built for each given posture/activity group. A more recent evolution of this system (using insoles instead of sensorized shoes) is presented in [34].

## 3. Methodology

**Figure 1:**
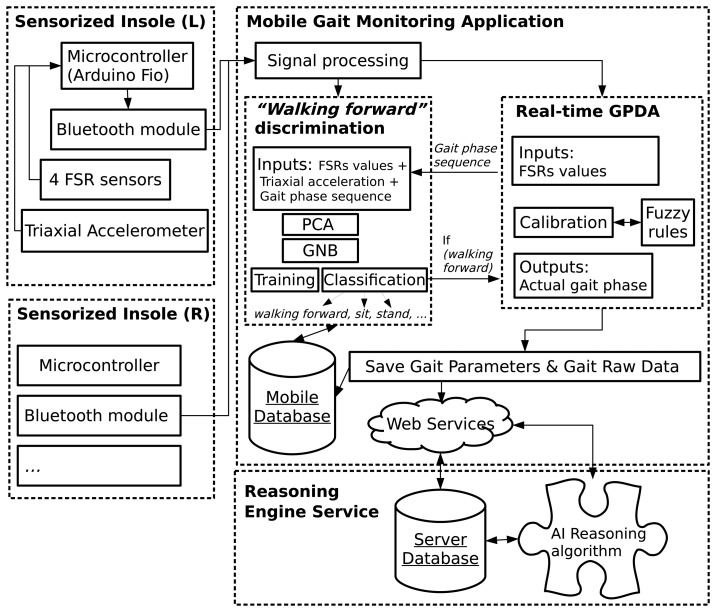
Overview of the proposed system for continuous gait monitoring.

Our system comprises three main modules. Each module is defined as a set of hardware and software elements with a specific purpose in the ambulatory system under development. An overview of the system is shown in Figure 1.

The first module (boxes on the left in Figure 1) describes the hardware and software co-design of each sensorized insole. On the hardware level, each insole is an assembly consisting of two kinds of sensors: the force-sensitive resistors (FSRs) and the inertial measurement unit (IMU). In order to manage data acquisition, one Arduino Fio microcontroller [35] is programmed with a piece of software to provide the Bluetooth module with the data from the sensors. The Bluetooth module (set to slave mode) establishes the communication between the insole module and the mobile application module.

The mobile gait monitoring application (the top right box in Figure 1) is the system's main software component. This module essentially runs in a smartphone's background service and includes our solution's two main algorithms (represented as submodules). The first is the fuzzy rule-based inference algorithm for real-time gait phase detection (GPDA), which will be explained in detail in Section 3.2. The second algorithm allows us to include the continuous monitoring functionality in the system by means of PCA for feature selection and the GNB classifier for foot movement pattern recognition (walking forward discrimination). It will be explained in detail in Section 3.3. Essentially, when the walking forward action is recognized, the gait phase detection will continue to capture gait parameters until the action finishes.

The gait phases and other estimated gait parameters will be uploaded to a remote server (reasoning engine module) (the bottom box in Figure 1), where reasoning techniques will be applied for different purposes (e.g., diagnosis of frailty or pre-dementia status).

### 3.1. Hardware and Software Description

**Figure 2:**
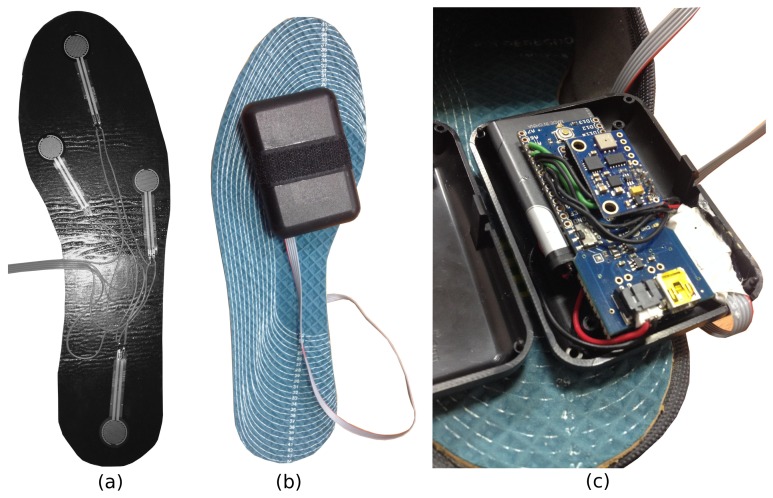
Insole hardware prototype. Arrangement of FSR sensors on the insole (**a**). Final prototype (**b**). Arduino Fio + 9DOF IMU (GY-80) + 3.7V LiPO batt. + Bluetooth module WLS125E1P (**c**).

Figure 2 details the hardware components in each insole. The arrangement of FSRs is illustrated in Figure 2a. Each FSR sensor consists of a conductive polymer, which changes its resistance in a predictable manner following the application of force to its surface. The harder the force, the lower its resistance. When no pressure is applied to the FSR, its resistance will be larger than 1 MΩ. The FSRs used here have a round 0.5″ diameter sensing area (provided by Sparkfun [36]). Four FSR sensors are placed on each insole. The first sensor is located at the hallux (toe); two more sensors are located at the forefoot (first and fifth metatarsals); and one more is located at the heel.

In Figure 2b, the final prototype is illustrated. The microcontroller, the 9 DOF IMU, the Bluetooth module, one resistor array and a 3.7-V LiPo battery are encapsulated inside a small plastic box, which will be attached to the posterior part of the tibia, near the Achilles tendon. The 9 DOF IMU, named GY80, is the small board on top of the Arduino Fio (Figure 2c). It is a popular device that can connect to the Arduino through the I2Cprotocol. There are four sensors on it, a gyroscope (L3G4200D), an accelerometer (ADXL345), a magnetometer (HMC5883L) and a barometer and temperature sensor (BMP085).

The signals from the FSRs and from the IMU (only the triaxial accelerometer is used here) are preprocessed by the microcontroller and packed to be sent to the smartphone by the Bluetooth module. In the built prototype, a Bluetooth Bee (WLS125E1P) device from Seeedstudio [37] is used for this task. The raw data from each insole are sent to the smartphone gait monitoring application at a sampling rate of 50 Hz. The application runs under the Android platform, using two separated threads to manage the connection with each insole and another thread to synchronize sensor events. Once the data are acquired, the raw data will be smoothed through a low pass filter to remove small fluctuations before feeding the two main algorithms detailed in Sections 3.2 and 3.3.

### 3.2. Real-Time GPDA

**Figure 3:**
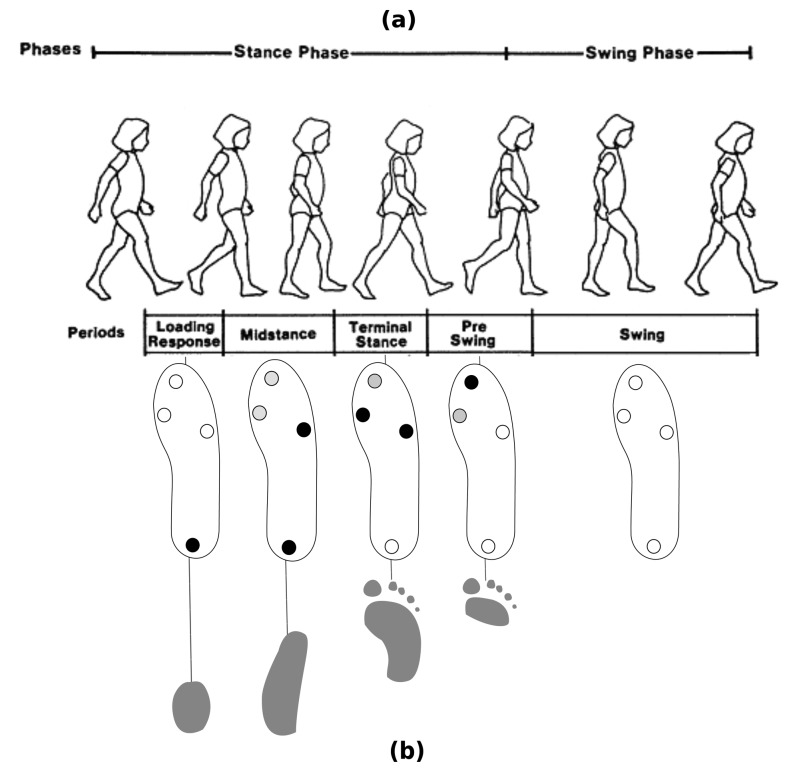
Detected gait subphases (**a**). FSR and foot pressure patterns (**b**).

The human gait cycle can be divided into two phases: stance and swing [1]. However, the complexity of human walking involves different kinetics and kinematics events within these phases. Therefore, a more refined classification is required to model the human gait cycle properly. Perry [1] distinguishes eight different subphases in her model of the human gait cycle. In our solution, derived from Perry's model, we segment five subphases (Figure 3a). The stance subphases (LR, MSt, TSt, PSw) can be identified only by examining the foot pressure patterns using the FSRs. However, our pressure sensors are useless for identifying Perry's proposed swing subphases (initial swing (ISw), mid-swing (MSw) and terminal swing (TSw). In our case, only the swing phase (Sw) as a whole can be detected when no pressure is exerted on any FSR. To detect the three swing subphases, a procedure involving two gyroscopes to estimate the knee angle (as in [30]) could be implemented; however, the device would become more intrusive.

#### 3.2.1. Relationship between Gait Phases and Foot Pressure Patterns

Figure 3b represents the expected pressure patterns in each stance subphase (LR, MSt, TSt, PSw). Four circles in each insole indicate the FSRs' positions. A black circle signifies a high force exerted on the related FSR. A white circle denotes a low pressure or no pressure is exerted; and finally, a gray circle indicates that FSR's signal is unused (or irrelevant) to describe the related gait phase through its pressure pattern. For example, in the mid-stance (MSt), force is still exerted on the heel; however, the center of pressure is moving towards the mid-foot, and depending on individual gait patterns, a small pressure can appear on the forefoot. Therefore, the toe and the first metatarsal status are not considered, but the fifth metatarsal and the heel are expected to experience high pressure.

#### 3.2.2. Fuzzification Process

As we stated in Section 2, gait phase detection based on thresholds or fixed rules has some limitations. In particular, the human gait cycle is not a set of discrete events; rather, the transitions between foot pressure patterns are soft and continuous. Fuzzy logic is able to capture the continuity and smoothness involved in human walking by means of an adequate number of membership functions and the appropriate choice as to their nature. Therefore, if we again pay attention to Figure 3b, we can express each of the stance subphases as human-readable rules. For example, we can model the beginning of the TSt phase as follows:

(1) $$\text{If}\;{\text{FSR}}_{\text{heel}}\;\text{is}\;\text{low}\;\text{AND}\;{\text{FSR}}_{5th}\;\text{is}\;\text{high}\;\text{AND}\;{\text{FSR}}_{1st}\;\text{is}\;\text{high}\Rightarrow \text{TSt}$$

where *FSR_heel_* represents the FSR placed on the heel and *FSR*_5_*_th_* and *FSR*_1_*_st_* the FSRs placed on the fifth and first metatarsals, respectively. As expected, there are no conditions about the FSR on the toe (hallux); regardless of its low or high value, if the other requisites are satisfied, the gait cycle reaches the terminal stance phase.

Table 3 represents the expected input levels for each rule's activation, according to the foot pressure patterns shown in Figure 3b. All of the stance subphases are modeled, as well as the swing phase.

A fuzzy approach similar to the one presented by Kong and Tomizuka in [38] is adopted here with some divergences. Two different fuzzy sets (low and high) define each linguistic variable (*FSR_x_*). As in [38], only one rule models each stance subphase and the swing phase. However, there are some differences in how the rules are constructed and in the activation levels of some of the inputs.

At this point, an obvious question arises: how do we decide whether an FSR input has more membership in the “low” status category or the “high” one?; or in other words, how is each fuzzy set constructed? To answer this question, two membership functions define each linguistic input variable's domain. These membership functions have been carefully chosen to preserve the principles of continuity and smoothness over their entire range, as well as ensuring continuity and smoothness in the transitions between gait phases. Therefore, two normalized symmetric sigmoid functions are used to represent the grade of membership for low status (Equation (2)) and the grade of membership for high status (Equation (3)) of an input value:

(2) $$f_{\text{low}}\left(x\right)=\frac{1}{\left(1+e^{s\left(x-x_{0}\right)}\right)}$$

(3) $$f_{\text{high}}\left(x\right)=1-f_{\text{low}}\left(x\right)$$

where *x* represents the input value from each sensor, normalized in [0, 1]; *x*_0_ is the inflection point where the grade of membership is exactly 0.5 (it will be fixed as *x*_0_ = 0.5 to maintain symmetry in the domain of the input variable); and *s* represents the slope of the sigmoid function.

**Figure 4:**
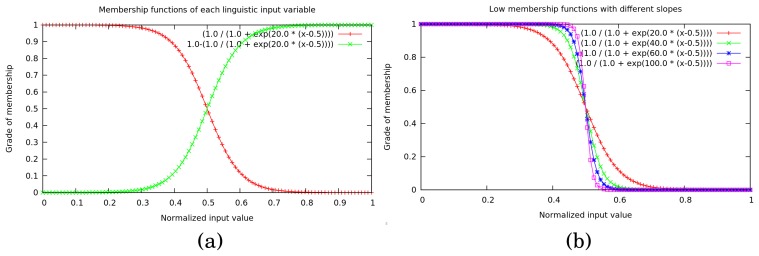
Membership functions of each linguistic input variable (**a**). Low membership functions with different slopes (**b**).

Figure 4a shows an example of the two membership functions' final configuration in a linguistic input variable (*FSR_x_*). The red function represents the grade of membership in the low fuzzy set and the green the grade of membership in the high fuzzy set. Across the domain, the sum of memberships is always 1. It is important to note the influence of the chosen slope. As *s* increases (*s* → ∞), the membership function becomes clearer, and transitions lose smoothness, although they are faster. Figure 4b shows four sigmoids with different slopes (from 20.0 to 100.0).

After some empirical trials using the system, a slope of *s* = 15.0 was chosen for all membership functions, except the sigmoid functions in the *FSR_toe_* variables. In these cases, a slope of *s* = 50.0 was used. The main reason for this exception lies in the fact that forefoot pressure (particularly toe pressure) during the PSw phase is very short, and the transition between non-pressure and pressure patterns occurs more sharply. Therefore, the PSw phase is better segmented using this slope configuration.

One important aspect prior to the fuzzification relates to the signal processing of the sensor data before feeding the GPDA. This step is described in the overview of the system in Figure 1. The signal from every sensor is smoothed through a simple low pass filter before being used by the walking forward discrimination and the real-time GPDA submodules. Additionally, each input value must be normalized in [0, 1] to be in the same domain as the related sigmoid function (see Figure 4a). We have developed a calibration procedure to be performed the first time a subject uses the insoles or if he/she changes footwear. The calibration process is also reflected in the real-time GPDA submodule in Figure 1. It involves two stages for each foot:

1. Lifting the foot off the ground and measuring the forces exerted on each FSR (no weight is applied).
2. Posing the whole foot down and remeasuring the forces exerted again (full body weight is applied).

**Figure 5:**
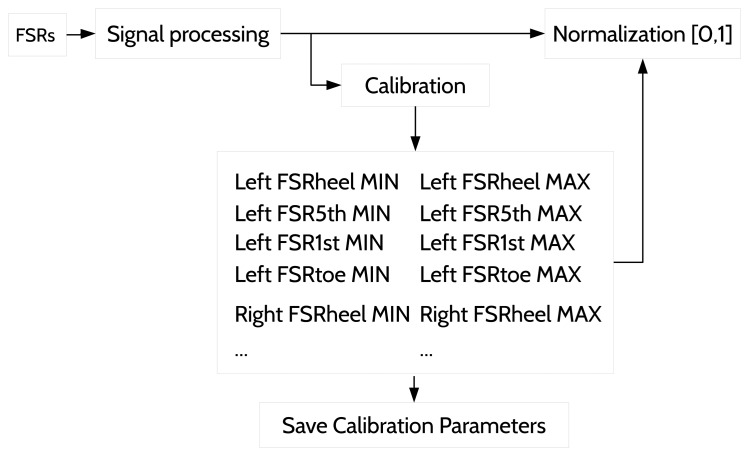
Sensor value normalization.

The calibration process is performed to estimate the effective range of each FSR [*min*, *max*]. The retrieved data in each stage are sorted, and the first quartile is calculated and assigned to the min (first stage) and to the *max* (second stage), respectively. Once the calibration ends, the estimated *min* and *max* values for each input variable are used continuously in the normalization process. (Figure 5).

The estimation of the effective range of each FSR input is necessary, because some FSRs could attain a light pressure even when the foot is in the air (0.5 to 1.0 V instead 0.0 V) due to the contact of the FSRs with the foot. On the other hand, the 3.3 V expected at maximum pressure normally are around 2.8 to 3.0 V and vary from one FSR to other.

Returning again to the example shown in Equation (1) and the rules outlined in Table 3, the determination of each gait's phase output membership requires an inference operator. Each AND intersection in Equation (1) can be interpreted as a T-norm operator. Kong and Tomizuka in [38] use the algebraic product as the T-norm; in our case, however, we implement the minimum T-norm. Based on the definition rule of the TSt phase from Equation (1), if the following membership grades are given as an example:

- The grade of membership for low is 0.95 in *FSR_heel_*, *FSR_heel_*(*x_low_*) = 0.95
- The grade of membership for high is 0.90 in *FSR*_5_*_th_*, *FSR*_5_*_th_*(*x_high_*) = 0.90
- The grade of membership for high is 1.00 in *FSR*_1_*_st_*, *FSR*_1_*_st_*(*x_high_*) = 1.00

the output grade of membership for TSt is: *min*{0.95, 0.90,1.00} = 0.90.

As expected, if all of the conditions are satisfied, the output tends to 1.

### 3.3. Activity Recognition: Walking forward Discrimination

In this section, the second main component of our solution is detailed. Looking again at the overview of the system described in Figure 1, this section corresponds to the “walking forward” discrimination algorithm, inside the mobile gait monitoring application.

Once the submodule for detecting gait phases in real-time is implemented, the workflow must focus on the algorithm for gait recognition. Specifically, the ideal is achieving nearly real-time classification of several foot movement patterns. The proposed algorithm is based on three stages:

1. Feature extraction (Section 3.4):
  - From the FSR signals (in the time and frequency domains).
  - From the anterior-posterior acceleration (forward-backward accel. → *Z* axis) (in the time and frequency domains).
  - From the vertical acceleration (up-down accel. → *Y* axis) (in the time and frequency domains).
  - From the gait phase sequence; it uses the last estimated gait phases from the GPDA as input; it will be further explained later.
2. Feature selection (Section 3.5): Based on the PCA algorithm for dimension reduction.
3. Classification of foot movement patterns (Section 3.6): by means of the GNB classifier, the system is able to predict eight low-level activities or foot movement patterns:
  - walking forward, walking backwards, lateral walking (walking left), lateral walking (walking right), turning left, turning right, sitting down and standing up.

The classification of foot movement patterns enables making a distinction between the walking forward activity and the other foot actions, which are useless for gait phase detection. When the GNB classifier identifies a straight-line gait, it automatically notifies the GPDA submodule, and the latter begins to store the gait phases in a new gait instance until the walking forward activity finishes. In this way, controlled and continuous gait monitoring is achieved, and the system is capable of identifying gait phases in real time, without any user interaction and throughout the day.

It is important to point out that when the duration of the walking forward activity is too short (e.g., a sequence of one or two strides), the proposed solution has insufficient sensitivity to fully identify the walking forward sequence. Generally, the classifier takes two seconds to recognize the foot movement pattern. Regardless, we are not interested in detecting these kinds of walking forward sequences. That is, if the aim is to provide a continuous gait monitoring solution throughout the day, it is not necessary to capture every isolated step. In fact, excessively short gait sequences are inadequate to study gait variability. In the same way, the first steps at the start of a walking forward sequence do not properly represent the subject's gait until stability is enhanced. Furthermore, when the sequence is near its end, the gait slows down until stopping. Therefore, if a reasoning engine will use extracted gait instances, neither the beginning of the walking forward sequences nor the end should be considered.

### 3.4. Feature Extraction and Training Dataset

The GNB classifier is trained with 8 sets of 1200 samples, one for each low-level activity recognized. For the time being, all of the training sets are carried out by the same test subject with a normal gait.

As indicated previously, some of the extracted features are in the time domain and others in the frequency domain. The fast Fourier transform (FFT) is used to transform the required data to the spectral domain. The buffer size of each FFT is 128 values (corresponding to ∼2.5 s due to the system runs at 50 Hz). Variations in each FFT's buffer size could provide different results and deserve exploration. Nevertheless, buffer sizes between 2 and 3 s are sufficient to characterize the foot movement pattern in a real-time situation with a reasonable delay.

Each received sample primarily consists of the eight FSR values (four per insole), the antero-posterior and vertical accelerations, the current gait phase in each foot and the timestamp related to the sample. Eight buffers contain the most recent 128 values associated with each FSR sensor, and four buffers (two per insole) contain the most recent 128 values of the antero-posterior and vertical accelerations, respectively.

**Figure 6:**
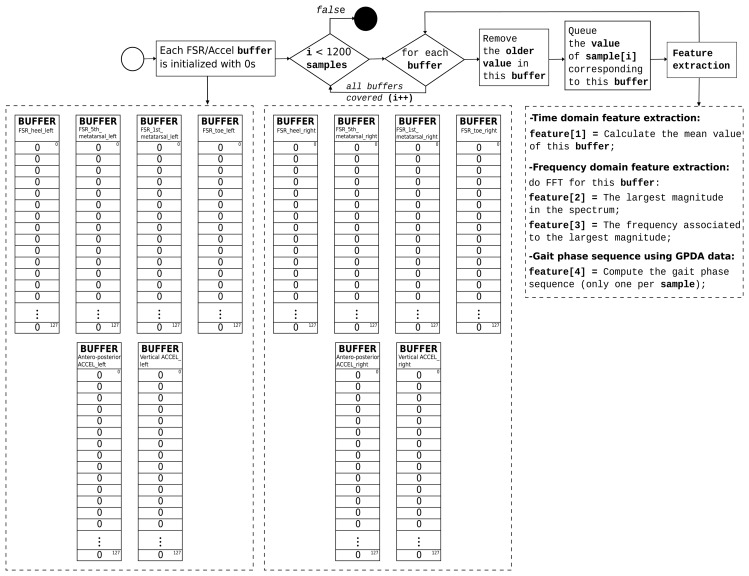
Feature extraction for each low-level activity (set of 1200 samples).

The feature extraction is explained in the flow diagram in Figure 6.

**Figure 7:**
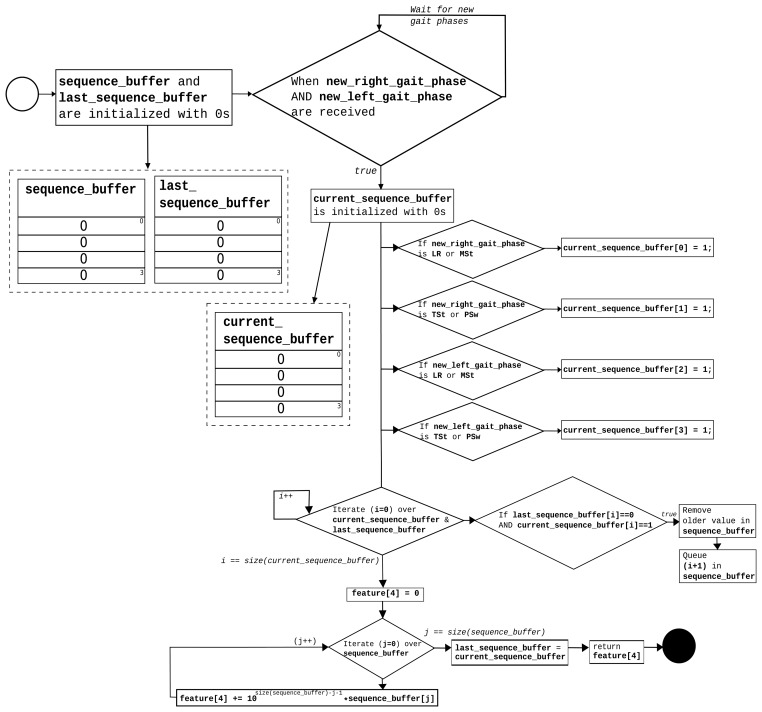
Feature 4: Computing the gait phase sequence.

For each sample, 37 features are extracted. Features 1–3 are calculated for each FSR and acceleration buffer. Feature 4 (gait phase sequence) is computed once per sample. The process is described in the flow diagram in Figure 7.

Feature 4 attempts to characterize the “walk forward” and “walk backwards” movement patterns that are taking advantage of the gait phase activation sequence, which is provided by the GPDA submodule. The sequence_buffer stores the last four activated phases. Finally, a single natural number in base 10 is composed using this buffer, and it is provided as Feature 4.

### 3.5. Feature Selection: PCA for Dimension Reduction

The PCA's desired outcome is to project the feature space (our dataset consists of 37 × 9600 dimensional (37 features per sample and 1200 samples for each of the 8 foot movement patterns or low-level activities recognized (9600 samples ignoring class labels)) samples) onto a smaller subspace that represents our data “well” (with minimal loss of information). To achieve this goal, the first step is computing the 37-dimensional mean vector using all of the samples:

(4) $$m=\frac{1}{n}\sum_{j=1}^{n}x_{j}$$

where *x_j_* is the *j* sample of the dataset and *n* = 9600 is the size of the dataset containing all samples, ignoring divisions in classes.

The next step is to compute the scatter matrix (*S*), using the samples of the dataset and the mean vector (Equation (4)) with the following equation:

(5) $$S=\sum_{j=1}^{n}\left(x_{j}-m\right){\left(x_{j}-m\right)}^{T}$$

In this case, the scatter matrix is a 37 × 37 matrix that allows us to calculate the eigenvectors and eigenvalues. Some of the eigenvectors will form the axes of the new feature subspace. However, the eigenvectors only define the directions of the new axes (they have all the same length of 1). In order to decide which eigenvectors will compose the final lower-dimensional subspace, it is necessary to pay attention to the eigenvalues. The approach involves choosing the top *I* eigenvectors with the largest eigenvalues, which own more information about the data distribution, and dropping the rest. Concatenating these *I* eigenvectors, a transformation matrix (*W*) is composed. *W* is a 37 × *I*-dimensional matrix, where *I* < 37.

Finally, we transform the samples onto the new *I*-dimensional subspace using *W* via Equation (6):

(6) $$y=W^{T}\times x$$

As we will show in Section 4.2, after several trials, we get the best accuracy (∼92%) in classification where *I* = 22.

### 3.6. Classification of Foot Movement Patterns Using the GNB Classifier: Walking forward Discrimination

A naive Bayes classifier is trained using the dataset and the selected features (*I*) from the PCA. Based on the Bayes' formulae, the posterior probability is determined by:

(7) $$p\left(W=w_{k}|X=x_{1j},\cdots ,x_{Ij}\right)=\frac{p\left(W=w_{k}\right)p\left(X=x_{1j},\cdots ,x_{Ij}|W=w_{k}\right)}{p\left(X=x_{1j},\cdots ,x_{Ij}\right)}$$

where *p*(*W* = *w_k_*) represents the prior probability, defined as the general probability of encountering the *w_k_* class (*i.e.*, walking forward) and *p*(*X* = *x*_1_*_j_*, ⋯, *x_Ij_*) is the evidence factor of the given sample we want to classify into one of the (*K* = 8) foot movement patterns or classes. Each symbol *x_ij_* refers to the value of the *i*-th feature in the *j*-th sample. Finally, *p*(*X* = *x*_1_*_j_*, ⋯, *x_Ij_*|*W* = *w_k_*) is the likelihood of *w_k_* with respect to *X*. The classifier will predict that *X* = *x*_1_*_j_*, ⋯, *x_Ij_* belongs to the class with the highest posterior probability.

As *p*(*X* = *x*_1_*_j_*, ⋯, *x_Ij_*) is the same for all of the *K* classes and only works as a scale factor that guarantees that the posterior probabilities' sum is unity, it can be discarded. Consequently, only the enumerator (*p*(*W* = *w_k_*)*p*(*X*|*W* = *w_k_*)) needs to be maximized. We can also assume that prior probabilities are equally likely, that is *P*(*W* = *w*_1_) = *P*(*W* = *w*_2_) = ⋯ = *P*(*W* = *w_k_*), and therefore, we maximize only the likelihood of *w_k_* respect to *X*, which is quite simple, yet powerful:

(8) $$p\left(W=w_{k}|X=x_{1j},\cdots ,x_{Ij}\right)\propto \underset{w_{k}}{\arg\max}p\left(X=x_{1j},\cdots ,x_{Ij}|W=w_{k}\right)$$

The maximization process can be further simplified by making the naive assumption of class conditional independence:

(9) $$p\left(W=w_{k}|X=x_{1j},\cdots ,x_{Ij}\right)\propto \underset{w_{k}}{\arg\max}\prod_{i}^{I}p\left(X=x_{ij}|W=w_{k}\right)$$

As every *x_ij_* is a continuous value, we can assume that each feature value within a class follows a Gaussian distribution:

(10) $$p\left(X=x_{ij}|W=w_{k}\right)=\frac{1}{\sigma_{ik}\sqrt{2\pi }}\exp\left[-\frac{1}{2}{\left(\frac{x_{ij}-\mu_{ik}}{\sigma_{ik}}\right)}^{2}\right]$$

The maximum likelihood estimation (MLE) technique is used to get the best estimates of mean (*μ_ik_*) and variance (*σ_ik_*) in order to model the density function from the given training samples of each class:

(11) $$\ln\left(p\left(W=w_{k}|X=x_{1j},\cdots ,x_{Ij}\right)\right)\propto \underset{w_{k}}{\arg\max}\sum_{i}^{I}\ln\left(p\left(X=x_{ij}|W=w_{k}\right)\right)$$

(12) $$\ln\left(p\left(W=w_{k}|X=x_{1j},\cdots ,x_{Ij}\right)\right)\propto \underset{w_{k}}{\arg\max}\sum_{i}^{I}\ln\left(\frac{1}{\sigma_{ik}\sqrt{2\pi }}\right)-\frac{1}{2}{\left(\frac{x_{ij}-\mu_{ik}}{\sigma_{ik}}\right)}^{2}$$

(13) $$\begin{array}{cc} \Rightarrow {\hat{\mu }}_{ik}=\frac{1}{n}\underset{j}{\text{∑}}x_{ij}, & {\hat{\sigma }}_{ik} \end{array}=\frac{1}{n}\sum_{j}{\left(x_{ij}-{\hat{\mu }}_{ij}\right)}^{2}$$

The normal distribution parameters (*μ̂_ik_*, *σ̂_ik_*) are estimated for each *i* feature using all of the samples (*j* = 1 … 1200) in each *k* class or foot movement pattern (Equation (13)). Subsequently, the estimated parameters are used in Equation (12). The GNB classifier outputs the class with the largest value (proportional to its probability).

## 4. Evaluation

### 4.1. Evaluation of the Real-Time GPDA Submodule

**Figure 8:**
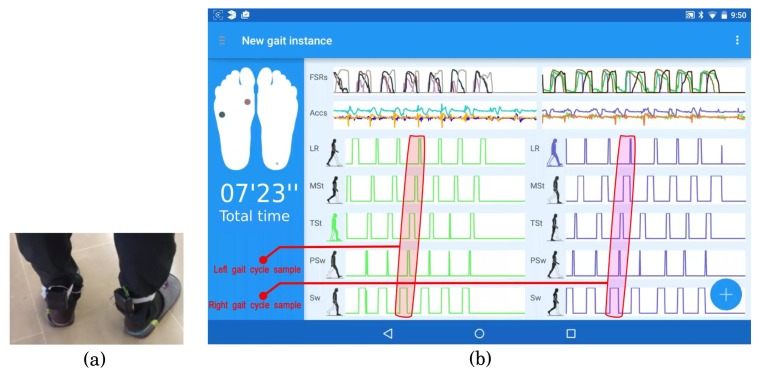
Wireless sensorized insoles setup (**a**) and new gait instance user interface (**b**).

To verify the gait phase detection algorithm's accuracy, the system was tested on five male subjects. All candidates presented a healthy gait and had an age range of 33 ± 2. Prior to the gait data capture, the subjects were asked to perform some trials of walking with the ambulatory system attached as in Figure 8a. For each subject, three separate trials were stored, each of them consisting of 10 gait cycles for each foot. The first and the last gait cycles in the trials were discarded. As we said in Section 3.3, the start and the end of the walking forward sequence do not properly represent the subject's gait, due to poor stability (in the case of the beginning) and gait deceleration (at the end of the walking sequence).

For each valid cycle in each trial, the five gait phases of normal gait (LR, MSt, TSt, PSw, Sw) were properly identified in the expected order (one phase consecutively after another and demarcated with the maximum membership ∼1).

In Figure 8b, a screenshot shows the user interface of the new gait instance dialog, which displays the segmented gait phases in real time during an explicit gait capture action. The drawn feet in the left column reflect the pressures registered by the FSR sensors using colored circles (darker when more pressure is exerted). The main frame on the right displays the FSR signal magnitudes and triaxial accelerations in each insole (top), as well as the membership outputs to each gait phase (bottom). The screen capture in Figure 8b reflects the beginning of a gait trial, with seven cycles/strides collected. The highlighted areas in red enclose an example of gait cycle for the left foot and for the right foot, respectively.

To perform a more thorough analysis of the gait phase detection algorithm's accuracy, the detected gait phases from the trials were compared with a theoretical model of human gait [1] generally accepted by the experts and used as a ground truth. The theoretical model of human gait proposed by Perry [1] was chosen rather than an empirical reference system based on a specific lab setting or a commercial solution for motion and gait analysis (such as Vicon systems or the GAITRite electronic walkway). The theoretical model provides a good approximation of the system's accuracy during gait phase detection, avoiding possible inaccuracies from the laboratory instrumentation.

For each gait trial, the average time of each gait phase was computed by the GPDA submodule (taking into account the eight valid cycles for the left and right feet separately) and compared with the expected time for this particular phase in the reference system. According to the human gait cycle proposed by Perry [1], the loading response (LR) occupies 10% of the total length of the cycle, mid-stance (MSt) 20%, terminal stance (TSt) also 20%, pre-swing (PSw) 10% and, finally, swing (Sw) the remaining 40% of the cycle time. For the ease of comparison, the percentages of Perry's gait phases are transformed into time units via Equation (14):

(14) $$\text{GPhaseTime}=\frac{\text{GPhase}\%}{100}\times \text{GCycleTime}$$

where *GPhaseTime* is the duration of the gait phase in the reference system, taking into account the defined percentage for this phase (*GPhase*%) and the duration of the actual gait cycle (*GCycleTime*) being measured.

Tables 4 and 5 exhibit the relative differences (in milliseconds) between the average duration of each gait phase estimated by the GPDA and the expected duration from the reference system. The values are computed for each trial. A negative average duration denotes a shorter detected gait phase relative to the reference gait phase. Conversely, a positive average value denotes a longer detected gait phase relative to the reference gait phase. Table 4 shows the relative differences associated with the left gait cycles and Table 5 the relative differences associated with the right gait cycles. The last row in each table contains the total average duration of each gait phase (Avg), as well as the standard deviation (SD). As can be seen, the larger differences from the reference system are in the MSt phases (overestimated around 78 ± 41 ms) and in the PSw phases (underestimated around −59 ± 19 ms). In terms of accuracy, the relative differences from the reference system are very similar for the left and right gait cycles.

### 4.2. Evaluation of the GNB Classifier for Foot Movement Pattern Recognition

To estimate the GNB classifier's accuracy, a six-fold cross-validation is performed. Each set of 1200 samples, one for each low-level activity recognized, is divided into six parts. In each of these six iterations, one part is used for testing and the remaining five for training. To analyze how the PCA helps improve the GNB classifier's estimated accuracy, we have tried different configurations that vary the number of selected features from the feature space (following the ranking of features imposed by the PCA algorithm, choosing those features that own more information about the distribution of the data) between a minimum of five and a maximum of 37 selected features.

**Figure 9:**
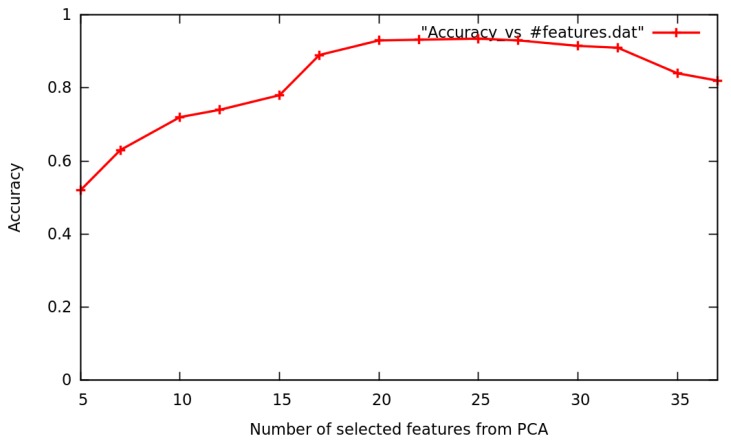
GNB classifier's accuracy depending on the number of selected features from PCA.

In Figure 9, the GNB classifier's accuracy during cross-validation analysis is plotted (y-axis) against the number of selected features (x-axis). The GNB classifier's accuracy follows a line of growth until a total of 22 features are selected, then holds a constant value around 90% to 92% accuracy and finally begins to decrease at 30 to 37 features (to 80% to 82% accuracy).

Applying cross-validation analysis to the eight training sets of 1200 samples, we can see how the GNB classifier's accuracy is enhanced by ten percent (reaching 92%). At the same time, the feature space is reduced from 37 to 22 dimensions.

### 4.3. Experimental Case: Non-Walking forward Detection

**Figure 10:**
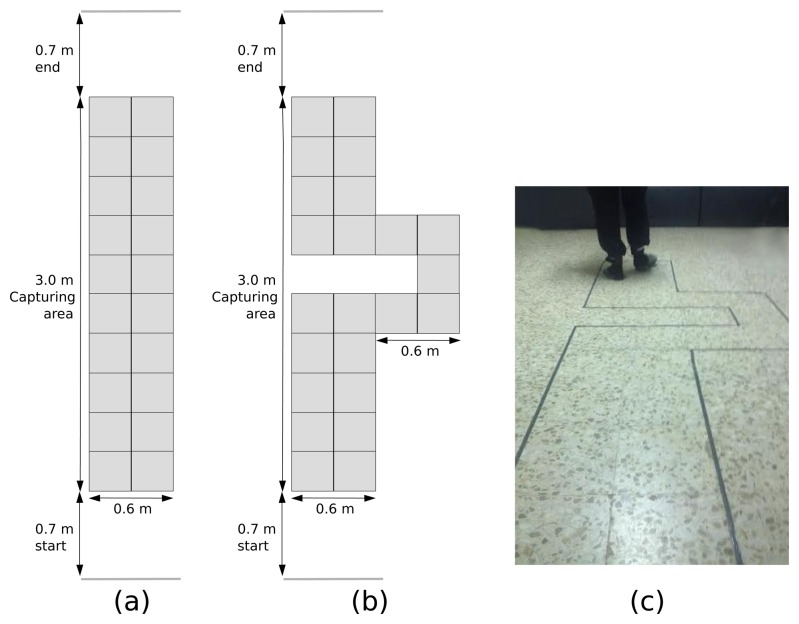
Straight-line path (**a**). Turning path (**b**). During the experiment (**c**).

We conducted a simple experiment to test the GNB classifier's ability to detect when the walking forward pattern is replaced by one of the other seven recognized foot patterns. Two gait paths were designed and painted on the ground to accomplish this task: first, a three meter-long straight-line path (Path A, Figure 10a), where only walking forward is expected to be detected; second, a path where a turn must be negotiated (Path B, Figure 10b).

The percentages of recognition of each foot movement pattern are illustrated in Table 6. Ten trials were performed by a test subject (31 years old and normal gait) in each path. The percentages were computed as the sum of the time a foot movement pattern was recognized between the trial's duration.

“Start” and “stop” lines in each path were placed 0.7 m in front of and past the capturing area to avoid the standing up detection at the beginning and at the end of the walking sequence and to ensure the subject had a comfortable speed when he reached the capturing area.

If we look at the percentages of recognition of each foot movement pattern in Path A (straight-line path), the walking forward pattern was detected an average of 99.64% ± 0.46% of the trial's duration, which represents that quantitatively, the walking forward action was recognized almost all of the time with only a few false negatives: 0.01% ± 0.03% (turning left) and 0.34% ± 0.45% (standing up). Additionally, there was a decrease in the walking forward percentage of recognition in Path B due to the turning actions. In this case, the walking forward pattern was detected an average of 80.45 ± 0.46, about 20% less than in the trials in Path A. The required turning actions to follow Path B made the turning left and turning right actions be recognized with average percentages of 10.53% ± 0.89% and 8.34% ± 1.50%, respectively. A few false positives were also identified: 0.01% ± 0.02% (left lateral walking) and 0.67% ± 0.43% (standing up). It is important to note that the turning actions were correctly recognized when they took place, because this fact cannot be reflected in the table. The influence of non-walking forward actions has been proven in this experiment. In this manner, the system can be used to identify a straight-line gait over time, which is the most useful for QGA.

## 5. Conclusions and Future Work

This paper introduces an approach to providing a wearable solution for continuous gait monitoring focused on gait phase detection. The implemented prototype consists of a pair of wireless insoles equipped with pressure sensors and inertial measurement units, which supply the required data to enable implementation of the system's two main functional components. First, the GPDA module allows real-time demarcation of stance subphases and the swing phase. Second, the GNB classifier identifies several foot movement patterns and allows the isolation of walking forward actions. In principle, these two components could provide independent functionalities (as explicit gait analysis and low-level activity quantification, respectively). However, the combination of them is what makes possible a continuous gait monitoring system that does not require any user interaction. In this article, both components are evaluated through independent experiments. The accuracy of the GPDA is verified and compared with a theoretical reference model, which provides a rough idea of the general accuracy of gait phase detection and illustrates the system's response between gait phase transitions. However, as part of future work, it is necessary to evaluate the GPDA module using a reliable device generally accepted by experts as a ground truth (e.g., GAITRite, Vicon motion system, *etc.*). This will allow a quantitative error analysis, which should better support the accuracy assertions.

One of the problems we encountered during the evaluation of the GPDA is derived from the availability of only a single prototype of the sensorized insoles during this early stage of the investigation. Furthermore, the insoles were made for a Size 43 (European), and the subjects who volunteered in the trials had sizes from 41 to 44. This could provide inaccuracies in pressure measurement, due to the fact that the FSR sensors could not be properly arranged in the hallux and metatarsals.

On the other hand, the combination of the PCA + GNB classifier has proven accurate enough (around 90% to 92%) for the person who conducted the training, and it is a valid configuration for continuous gait monitoring when it is not critical to capture every single step. Nonetheless, it would be adequate to analyze the behavior of the classifier in depth when bigger training sets are adopted for each foot movement pattern. Furthermore, as future work, it is necessary to test other more advanced classifiers to overcome the weaknesses of a single GNB.

In addition to carrying out further individual evaluations of the algorithms, making a full assessment focused on the integration between the two main modules (real-time GPDA and walking forward discrimination) is a primary requirement of future work. Furthermore, the system must be adapted from continuous gait monitoring to long-term gait monitoring. This means that some heuristic rules must be implemented to verify that the extracted walking sequences are valid in terms of time duration, correct temporal order between steps, *etc*. In such a context, it is also important to evaluate the behavior of the system under real-life conditions and environments (e.g., apartments with older adults in independent living conditions). In this manner, the long-term assessment will make it possible to capture inherent gait variability over time, which is an important requirement for diagnosing several disabilities or diseases, such as Parkinson, peripheral neuropathy, frailty, dementia, and so forth.

## Figures and Tables

**Table 1:** Relationship between gait phases detected by Crea *et al.* [25] and Perry's gait cycle phases [1].

**Crea *et al.* [25] Gait Phases**	**Perry [1] Gait Phases**
Stance 1 (St1)	Loading response (LR) and mid-stance (MSt)
Stance 2 (St2)	Terminal stance (TSt) and pre-swing (PSw)
Swing (Sw)	Initial swing (ISw), mid-swing (MSw), terminal swing (TSw)

**Table 2:** Main similarities between the gait phase detection algorithm (GPDA) in Pappas *et al.* [4] and the solution presented in this paper. FSR, force-sensitive resistor; GNB, Gaussian naive Bayes.

**Pappas *et al.* [4]**	**Solution Presented Here**
Real-time GPDA: Rule-based approach as a finite state machine.	Real-time GPDA: Fuzzy-rule based approach.
Phases: stance, heel off, swing, heel strike.	Phases: LR, MSt, TSt, PSw, Sw.
Sensorized insoles: 3 FSRs (heel, 1st and 4th metatarsals) + gyroscope.	Sensorized insoles: 4 FSRs (heel, 1st and 5th metatarsals, hallux). There is also a 9-DOF IMU, but it is not used by the GPDA.
GPDA running on a microcontroller.	GPDA core running on a smartphone.
Insensitive to non-walking activities through the transition conditions in the finite state machine's inherent restrictions.	Insensitive to perturbations caused by non-walking activities through the GNB classifier.

**Table 3:** Fuzzy rules set.

***FSRheel***	***FSR*_5_***_th_*	***FSR*_1_***_st_*	***FSR****_toe_*	**Gait Phase (Membership Value)**
high	low	low	low	LR → 1
high	high	-	-	MSt → 1
low	high	high	-	TSt → 1
low	low	-	high	PSw → 1
low	low	low	low	Sw → 1

**Table 4:** Average duration of each estimated gait phase *vs.* Perry's gait cycle (left cycles). Avg, average.

**Subjects**	**Trials**	**LR (ms)**	**MSt (ms)**	**TSt (ms)**	**PSw (ms)**	**Sw (ms)**
Subject A	Trial 1	10	115	13	−98	5
	Trial 2	−40	140	5	−86	−21
	Trial 3	−71	148	8	−97	20

Subject B	Trial 1	34	38	15	−42	−44
	Trial 2	12	39	−3	−61	−40
	Trial 3	59	29	5	-45	−46

Subject C	Trial 1	−14	101	−20	−50	8
	Trial 2	−35	99	20	−75	5
	Trial 3	−29	99	−11	−52	0

Subject D	Trial 1	23	34	−6	−56	−39
	Trial 2	32	37	11	−45	−43
	Trial 3	47	41	12	−50	−40

Subject E	Trial 1	−25	69	−18	−38	−9
	Trial 2	−29	84	−14	−48	−12
	Trial 3	−13	80	4	−44	−1

	**Avg (±SD)**	−3 (±36)	77 (±38)	1 (±12)	−59 (±19)	−17 (±22)

**Table 5:** Average duration of each estimated gait phase *vs.* Perry's gait cycle (right cycles).

**Subjects**	**Trials**	**LR (ms)**	**MSt (ms)**	**TSt (ms)**	**PSw (ms)**	**Sw (ms)**
Subject A	Trial 1	−32	134	9	−85	−14
	Trial 2	−5	121	14	−94	−1
	Trial 3	−47	139	2	−75	6

Subject B	Trial 1	18	17	21	−64	−30
	Trial 2	43	42	26	−40	−41
	Trial 3	48	29	17	−40	−38

Subject C	Trial 1	−30	97	15	−65	11
	Trial 2	−26	102	18	−51	8
	Trial 3	−23	104	7	−56	10

Subject D	Trial 1	48	38	27	−43	−39
	Trial 2	22	21	22	−60	−27
	Trial 3	43	34	18	−39	−39

Subject E	Trial 1	−25	91	18	−61	13
	Trial 2	−25	98	13	−55	12
	Trial 3	−22	100	19	−47	12

	**Avg (±SD)**	−1 (±33)	78 (±41)	16 (±7)	−58 (±16)	−10 (±22)

**Table 6:** Percentages of recognition of each foot movement pattern in the two paths.

**Paths**	**Trials**	**W.F. (%)**	**W.B. (%)**	**L.W.L (%)**	**L.W.R (%)**	**T.L. (%)**	**T.R. (%)**	**S.D. (%)**	**S.U. (%) ^1^**
Path A	Trial 1	100	0	0	0	0	0	0	0
	Trial 2	99.23	0	0	0	0	0	0	0.77
	Trial 3	100	0	0	0	0	0	0	0
	Trial 4	100	0	0	0	0	0	0	0
	Trial 5	99.09	0	0	0	0.04	0	0	0.87
	Trial 6	98.94	0	0	0	0	0	0	1.06
	Trial 7	100	0	0	0	0	0	0	0
	Trial 8	99.16	0	0	0	0.09	0	0	0.75
	Trial 9	100	0	0	0	0	0	0	0
	Trial 10	100	0	0	0	0	0	0	0

	**Avg (±SD)**	99.64 (±0.46)	0 (±0)	0 (±0)	0 (±0)	0.01 (±0.03)	0 (±0)	0 (±0)	0.34 (±0.45)

Path B	Trial 1	81.14	0	0	0	10.30	7.03	0	1.53
	Trial 2	77.45	0	0	0	11.19	10.84	0	0.52
	Trial 3	79.38	0	0	0	9.92	9.76	0	0.94
	Trial 4	85.18	0	0	0	8.97	5.75	0	0.10
	Trial 5	81.83	0	0	0	10.45	7.28	0	0.44
	Trial 6	79.92	0	0	0	11.90	7.87	0	0.31
	Trial 7	81.67	0	0.06	0	9.82	8.00	0	0.45
	Trial 8	78.21	0	0	0	11.37	9.36	0	1.06
	Trial 9	80.12	0	0.05	0	11.26	8.13	0	0.44
	Trial 10	79.60	0	0	0	10.16	9.37	0	0.87

	**Avg (±SD)**	80.45 (±2.17)	0 (±0)	0.01 (±0.02)	0 (±0)	10.53 (±0.89)	8.34 (±1.50)	0 (±0)	0.67 (±0.43)

1. W.F., walking forward; W.B., walking backwards; L.W.L., lateral walking left; L.W.R., lateral walking right; T.L., turning left; T.R., turning right; S.D., sitting down; S.U., standing up.
